# Otolaryngology Diagnoses at a Student-Run Community Clinic

**DOI:** 10.7759/cureus.70155

**Published:** 2024-09-25

**Authors:** Brian P Quinlan, Wilhelmina Tan, Orly Coblens, Brian McKinnon

**Affiliations:** 1 Department of Otolaryngology - Head and Neck Surgery, University of Texas Medical Branch, Galveston, USA

**Keywords:** community health, ent, free clinic, otolaryngology, public health, student-run clinic

## Abstract

Purpose

Compared to insured individuals who have established pathways for primary and specialist care, uninsured patients often delay seeking medical attention and are more likely to rely on emergency departments or community clinics for low-cost services. This study aims to identify the most prevalent otolaryngology (ENT) diagnoses at a free, student-run community clinic for uninsured patients. Additionally, it seeks to elucidate their management needs in order to inform strategies for enhancing support and care for this vulnerable population.

Methods

The electronic medical records for a free, student-run community clinic (St. Vincent's Hope Student Clinic in Galveston, Texas) were reviewed. Patient age, sex, chief complaint, date of medical encounter, and encounter diagnoses from April 2021 to May 2024 were recorded; medical diagnoses were then manually stratified into system categories and diagnostic subgroups. Diagnoses unrelated to ENT were excluded and likely represented secondary encounter diagnoses.

Results

A total of 38 patients were evaluated across 51 total ENT encounters. The average number of encounters per patient per year was 1.34 (1.05-1.63, 95% CI). The most common ENT category was throat (37%), and the most common individual diagnosis was balance problems (11% of total diagnoses).

Conclusion

The most common ENT system was the throat, followed by ear, nose, and other. Balance problems, reflux, and allergic rhinitis were the most common individual ENT diagnoses. The analysis of data collected for this study may help direct future care and resource allocation at this clinic.

## Introduction

According to data from 2023, approximately 7% or 25.3 million patients are uninsured in the United States [1]. While insured patients readily turn to their primary care provider or in-network healthcare facilities, uninsured patients are less likely to utilize health services [2]. When they require medical care, uninsured patients often look towards free or low-cost community resources to receive their care, of which many are affiliated with academic institutions. These free clinics are critical in allowing individuals to receive healthcare that they would otherwise be unable to procure [3]. Medical students frequently become involved with these institutions and play a critical role in staffing and organizing efforts. Reviews have shown that student-run health clinics result in positive health outcomes while providing valuable educational experiences for students [4].

Established in 1969 in Galveston, Texas, students from the University of Texas Medical Branch run a student clinic supported by faculty and residents, named St. Vincent's Hope Student Clinic, which provides free service to over 15,000 uninsured community members in need. In April 2021, the clinic established an otolaryngology (ENT) unit, servicing patients with ear, nose, and throat complaints. This clinic aims to fill a need for specialty services that underserved patients often struggle to receive [5].

Previous studies have provided epidemiological data detailing medical diagnoses of ENT within specific regional areas. However, at the time of article submission, no studies have comprehensively reported all medical diagnoses encountered in a dedicated, year-round, student-run, free community clinic. This absence of data signifies a dearth of knowledge regarding the most prevalent ENT medical conditions in this setting. This study aims to determine the most common diagnoses to reveal the prevalent demands of managing otolaryngological concerns among local uninsured patients to understand better how to support future clinical endeavors.

## Materials and methods

The electronic medical record (EMR) for the free, student-run community clinic - St. Vincent's Hope Student Clinic - was reviewed. Medical students at the John Sealy School of Medicine run this clinic on Tuesday and Thursday nights and Saturday mornings under the direct supervision of faculty and residents from the University of Texas Medical Branch at Galveston. The specialized ENT clinic is hosted on the third Thursday night of each month to treat patients with ear, nose, and throat concerns. All medical ENT encounters of Galveston community patients treated at this clinic from April 1, 2021, to May 31, 2024, were included in the analysis. Data were initially obtained from the EMR using Slicer-Dicer (Epic Systems Corporation, Verona, United States), an EMR query tool, and then were downloaded and further analyzed using Microsoft Excel (Microsoft Corp., Redmond, United States). Encounters not involving primary ENT diagnoses were excluded from the data set and likely represented secondary encounter diagnoses.

Where initial data was insufficient, the EMR was reviewed manually to enter supplemental data to finalize the data set. Fifty-one medical encounters met inclusion criteria, and patient age, sex, chief complaint, date of medical encounter, and encounter diagnoses were recorded. Medical diagnoses identified by the International Classification of Disease (ICD-10) codes were manually stratified into four system categories and 32 diagnostic subgroups for further analysis. This project was reviewed by the University of Texas Medical Branch Institutional Review Board (Galveston, Texas) before the initiation of the study and was approved as a non-regulated research activity. The study methodology followed the Strengthening the Reporting of Observational Studies in Epidemiology (STROBE) statement for observational studies.

## Results

From April 1, 2021, to May 31, 2024, a total of 38 patients were evaluated during 51 ENT-related medical encounters at the clinic (Table 1). About 34 of these encounters included more than one ENT visit diagnosis, and the average number of diagnoses per encounter was 2.86. On average, each patient had 1.34 encounters per year (95% CI: 1.05-1.63). Patient ages ranged from 31 to 75 years, with a mean age of 52.66 years. The gender distribution was relatively even, with males accounting for 45% of the cohort (n=17) and females for 55% (n=21).

**Table 1:** Demographic table outlining ENT medical encounters by sex and age ENT: Otolaryngology

Demographics of ENT encounters in free student-run clinic	
Total number of encounters (N)	51
Encounters per patient per year (N (95% CI))	1.34 (1.05-1.63)
Number of encounters with >1 diagnosis (N)	34
Total number of diagnoses (N)	146
Patient sex	
Female (N (%))	21 (55%)
Male (N (%))	17 (45%)
Patient age (mean)	52.66
Patient age, range (min-max)	31-75

**Figure 1:**
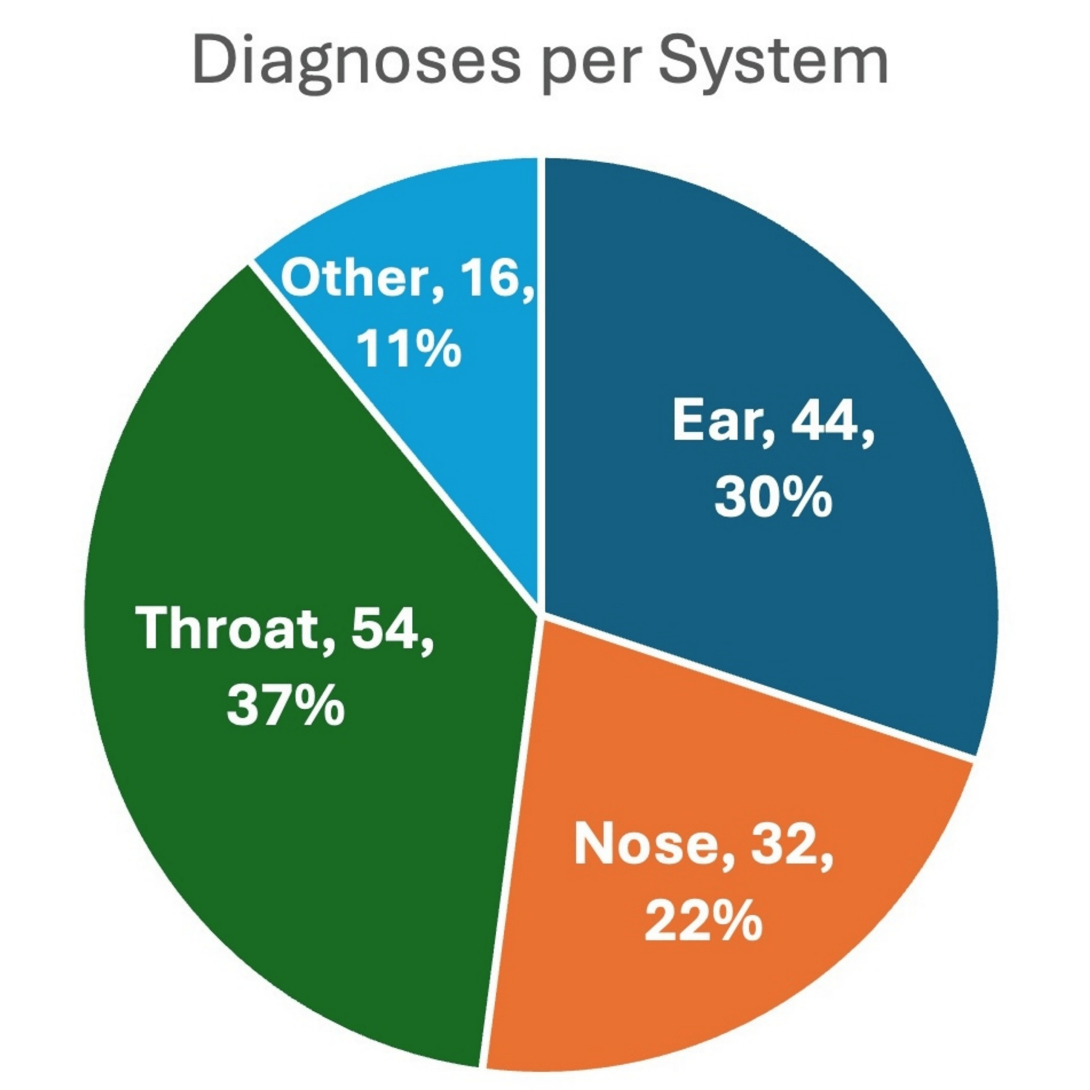
Medical encounter diagnoses were manually stratified into four systems and the frequency of each diagnosis per system by number and percentage is outlined above

The most frequently affected ENT system was the throat, which accounted for 37% of all diagnoses (n=54). This was followed by ear-related diagnoses at 30% (n=44), nose-related issues at 22% (n=32), and diagnoses in the "other" category at 11% (n=16) (Figure 1). Among throat diagnoses, the most common conditions were reflux (n=15, 28% of throat diagnoses), neck masses (n=8, 15%), and dysphonia (n=7, 13%). In the ear category, balance problems constituted 36% of ear diagnoses (n=16), followed by hearing loss at 14%(n=6) and both otalgia and tinnitus at 11% (n=5). In the nose category, allergic rhinitis was the most common diagnosis at 38% (n=12), followed by epistaxis at 16% (n=5) and nasal congestion at 16% (n=5). Temporomandibular joint (TMJ) syndrome was the only recurrent diagnosis in the "other" category, accounting for 50% (n=8) of those diagnoses.

**Figure 2:**
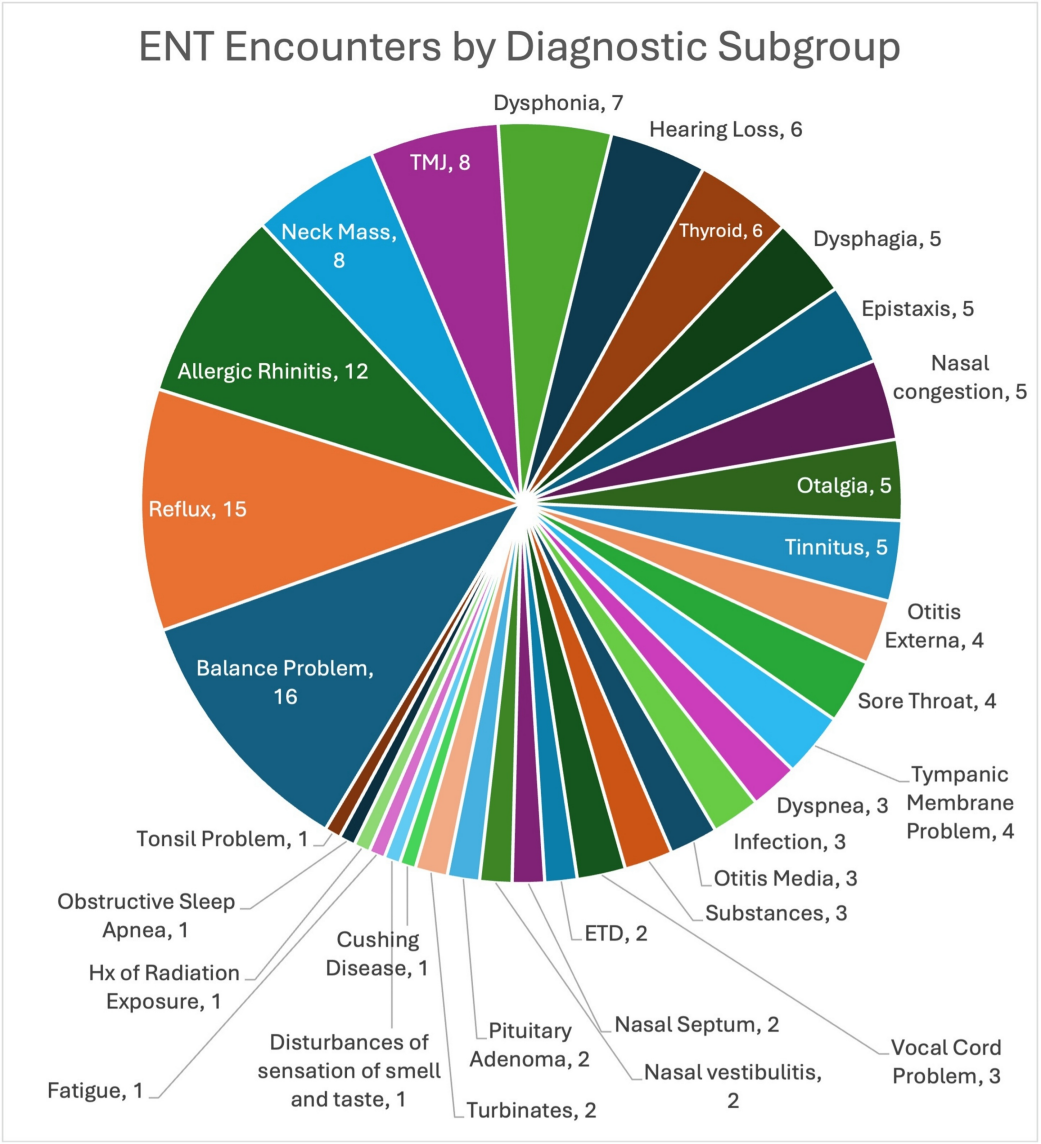
ENT encounter diagnoses were manually stratified into 32 diagnostic categories and the frequency of each diagnostic category by count is outlined above TMJ: Temporomandibular joint; ETD: Eustachian tube dysfunction; ENT: Otolaryngology

The most frequent diagnosis overall was balance problems with 16 cases (11% of all diagnoses), followed by reflux with 15 cases (10%) and allergic rhinitis with 12 cases (8%). The distribution of diagnoses by the system and the top diagnoses within each category are displayed in Tables 2-3 and Figure 2.

**Table 2:** Count of the diagnoses given as sorted into the 32 diagnostic subgroups TMJ: Temporomandibular joint; ETD: Eustachian tube dysfunction

Individual diagnosis category	Count (N (%))
Balance problem	16 (11%)
Reflux	15 (12%)
Allergic rhinitis	12 (8%)
Neck mass	8 (5%)
TMJ	8 (5%)
Dysphonia	7 (5%)
Hearing loss	6 (4%)
Thyroid	6 (4%)
Dysphagia	5 (3%)
Epistaxis	5 (3%)
Nasal congestion	5 (3%)
Otalgia	5 (3%)
Tinnitus	5 (3%)
Otitis externa	4 (3%)
Sore throat	4 (3%)
Tympanic membrane problem	4 (3%)
Dyspnea	3 (2%)
Infection	3 (2%)
Otitis media	3 (2%)
Substances	3 (2%)
Vocal cord problem	3 (2%)
ETD	2 (1%)
Nasal Septum	2 (1%)
Nasal vestibulitis	2 (1%)
Pituitary adenoma	2 (1%)
Turbinates	2 (1%)
Cushing disease	1 (1%)
Disturbances of sensation of smell and taste	1 (1%)
Fatigue	1 (1%)
History of radiation exposure	1 (1%)
Obstructive sleep apnea	1 (1%)
Tonsil problem	1 (1%)

**Table 3:** Most common diagnosis per ENT system The data has been represented as N (%). TMJ: Temporomandibular joint; ENT: Otolaryngology

Ear (N (%))		Nose (N (%))		Throat (N (%))		Other (N (%))	
1. Balance problem	16 (36%)	1. Allergic rhinitis	12 (38%)	1. Reflux	15 (28%)	1. TMJ	8 (50%)
2. Hearing loss	6 (14%)	2. Epistaxis	5 (16%)	2. Neck mass	8 (15%)		
3. Otalgia	5 (11%)	2. Nasal congestion	5 (16%)	3. Dysphonia	7 (13%)		
3. Tinnitus	5 (11%)						

## Discussion

Free community care clinics are essential as many individuals lack health insurance, which prevents them from accessing necessary healthcare services [6,7]. An ideal community clinic collaborates with various local resources and a hospital system capable of handling emergencies that require a higher level of care, ensuring that patients receive appropriate treatment. These clinics should alleviate the burden on emergency departments by proactively managing patient care and promoting preventive medicine [8,9]. However, the range of services offered at local care clinics is often constrained by the availability of volunteers in the community to staff the clinic and by financial limitations [10,11]. When a center is well-established, it can begin to offer more specialized care to patients; however, this poses a challenge as volunteers may have limited exposure to the specific health issues treated by these medical specialties.

This principle applies within the field of otolaryngology, which is not only specialized but also encompasses a wide range of sensitive treatment areas [12]. This study represents a novel effort to identify the most common diagnoses among the patient population in the Galveston County region, intending to enable clinic volunteers to become better prepared to provide specialized care to these individuals.

A significant finding is that neck masses account for 15% (n=8) of all ENT throat diagnoses analyzed. Despite the availability of flexible laryngoscopy in clinics, many patients often require case booking - a financial assistance program designed to facilitate access to more specialized care for further imaging studies. Among this population, many are ultimately diagnosed with head and neck cancer and require subsequent surgical resections or radiation treatments. It is essential to recognize that while many patients encountered in the student-run clinic present with benign conditions that are manageable either medically or procedurally, some will receive a diagnosis of malignancy and will need higher levels of care as well as additional financial assistance to ensure timely treatment and prevent delays that could worsen their prognosis [13].

The most relevant data for this study includes a 2023 investigation by Lukama et al., which examined the diagnostic accuracy and appropriateness of patient referrals for ENT specialist care at a resource-limited tertiary hospital in Zambia. In this study, 1,543 patients were reviewed, revealing that the most prevalent ENT diagnoses were allergic rhinitis, adenoid hypertrophy, chronic otitis media, acute tonsillitis, and laryngopharyngeal reflux [14]. The overlap of allergic rhinitis, chronic otitis media, and laryngopharyngeal reflux is consistent between this study and the findings of our analysis. However, Lukama et al. reported a significantly higher number of cases of adenoid hypertrophy and acute tonsillitis, which were less frequently observed at our community clinic. This discrepancy may be attributed to the differences in the primary settings studied - one being a clinical environment and the other a hospital setting - where surgical procedures cannot be performed at the community clinic. Although the results of these studies cannot be directly compared, the variations in diagnoses suggest that geographic region plays a significant role in the presentation of ENT concerns [15-17]. It is also important to note that St. Vincent's Hope Student Clinic patients must live in Galveston County and be uninsured to qualify as patients. These factors may lead to deviations from the most common ENT diagnoses typically observed within the field.

This data presents a unique opportunity to develop customized training programs for clinic volunteers, ensuring they are well-prepared to treat patients with otolaryngologic conditions. This is crucial as medical students constitute the primary staffing group at the clinic, and research has demonstrated that they benefit significantly from additional training in otolaryngology [18,19]. The authors recommend that to enhance otolaryngologic care at the St. Vincent’s Hope Student Clinic, students should participate in an orientation session as a prerequisite for approval as ENT-specific clinic volunteers. This session should cover the standard of care for balance disorders, gastroesophageal reflux, and allergic rhinitis, along with other prevalent diagnoses for each system, as previously outlined. We believe this initiative will enhance volunteer preparedness and their ability to address patient concerns effectively.

This data also enhances clinic leadership's understanding of the prevalent ENT needs within our community. By identifying the common conditions treated at the clinic, we gain valuable insights to guide the allocation of the clinic’s limited resources. Ultimately, we hope that by optimizing resource utilization, we can better support the financial needs of patients requiring more advanced care, such as those with malignant neck masses, as discussed earlier.

Limitations

The most notable weakness of this study is the small patient population sampled. While this study includes data from about three years, this period involves the conception of the clinic, which had a lower patient volume. Even after being established, the typical patient load is relatively small, with about three or four patients seen each clinic day. It is also noted that the frequency with which specific diagnoses present at the clinic is likely related to the structure and reach of the clinic and the care available in that setting. As such, any life-threatening or more severe diagnoses were unlikely to be seen in the analysis. Finally, the subjective nature of the authors’ manual categorization of specific diagnoses also must be considered as some diagnoses could be determined to be in more than just one category.

## Conclusions

Over the study period, 38 patients were seen at the clinic during 51 ENT-related encounters, with an average of 1.34 visits per patient per year. The most commonly affected system was the throat, followed by the ear, nose, and other systems. Balance problems, reflux, and allergic rhinitis were the most frequent individual diagnoses. The data from this study may help inform clinic leadership on resource allocation and volunteer training to better address the needs of patients with ENT-related issues.
